# Childhood tuberculosis is associated with decreased abundance of T cell gene transcripts and impaired T cell function

**DOI:** 10.1371/journal.pone.0185973

**Published:** 2017-11-15

**Authors:** Cheryl Hemingway, Maurice Berk, Suzanne T. Anderson, Victoria J. Wright, Shea Hamilton, Hariklia Eleftherohorinou, Myrsini Kaforou, Greg M. Goldgof, Katy Hickman, Beate Kampmann, Johan Schoeman, Brian Eley, David Beatty, Sandra Pienaar, Mark P. Nicol, Michael J. Griffiths, Simon J. Waddell, Sandra M. Newton, Lachlan J. Coin, David A. Relman, Giovanni Montana, Michael Levin

**Affiliations:** 1 Section of Paediatrics, Division of Infectious Diseases, Department of Medicine, Imperial College London, Norfolk Place, London, United Kingdom; 2 Department of Mathematics, Faculty of Natural Sciences, Imperial College London, 80 Queen's Gate, London, United Kingdom; 3 Department of Epidemiology and Biostatistics, School of Public Health, Imperial College London, Norfolk Place, London, United Kingdom; 4 Tygerberg Hospital, University of Stellenbosch, Cape Town, South Africa; 5 Red Cross War Memorial Children’s Hospital, University of Cape Town, Rondebosch, Cape Town, South Africa; 6 Institute of Infectious Diseases and Molecular Medicine, University of Cape Town, Cape Town, South Africa; 7 National Health Laboratory Service, Cape Town, South Africa; 8 Department of Clinical Infection, Microbiology and Immunology, Institute of Infection and Global Health, University of Liverpool, Liverpool, United Kingdom; 9 Brighton and Sussex Medical School, University of Sussex, Brighton, United Kingdom; 10 Department of Medicine, Stanford University School of Medicine, Stanford, California, United States of America; 11 Department of Microbiology and Immunology, Stanford University School of Medicine, Stanford, California, United States of America; 12 Veterans Affairs Palo Alto Health Care System, Palo Alto, California, United States of America; 13 Department of Biomedical Engineering, King's College London, London, United Kingdom; Karolinska Institutet, SWEDEN

## Abstract

The WHO estimates around a million children contract tuberculosis (TB) annually with over 80 000 deaths from dissemination of infection outside of the lungs. The insidious onset and association with skin test anergy suggests failure of the immune system to both recognise and respond to infection. To understand the immune mechanisms, we studied genome-wide whole blood RNA expression in children with TB meningitis (TBM). Findings were validated in a second cohort of children with TBM and pulmonary TB (PTB), and functional T-cell responses studied in a third cohort of children with TBM, other extrapulmonary TB (EPTB) and PTB. The predominant RNA transcriptional response in children with TBM was decreased abundance of multiple genes, with 140/204 (68%) of all differentially regulated genes showing reduced abundance compared to healthy controls. Findings were validated in a second cohort with concordance of the direction of differential expression in both TBM (r^2^ = 0.78 p = 2x10^-16^) and PTB patients (r^2^ = 0.71 p = 2x10^-16^) when compared to a second group of healthy controls. Although the direction of expression of these significant genes was similar in the PTB patients, the magnitude of differential transcript abundance was less in PTB than in TBM. The majority of genes were involved in activation of leucocytes (*p =* 2.67E^-11^) and T-cell receptor signalling (*p =* 6.56E^-07^). Less abundant gene expression in immune cells was associated with a functional defect in T-cell proliferation that recovered after full TB treatment (p<0.0003). Multiple genes involved in T-cell activation show decreased abundance in children with acute TB, who also have impaired functional T-cell responses. Our data suggest that childhood TB is associated with an acquired immune defect, potentially resulting in failure to contain the pathogen. Elucidation of the mechanism causing the immune paresis may identify new treatment and prevention strategies.

## Introduction

Children account for more than a million new cases of tuberculosis (TB) annually, with around 80 000 deaths each year [1, 2]. In children dissemination of *Mycobacterium tuberculosis* (*M*. *tuberculosis*) occurs more frequently than in adults and occurs soon after primary infection, resulting in extrapulmonary disease and infection of the brain, bones and other organs [2, 3]. Despite anti-mycobacterial treatment, mortality rates for children with disseminated disease such as TB meningitis (TBM) are 10–20%, and over 50% of survivors suffer long-term neurological deficits [1, 2].

Although a vast amount is known about the interaction of pathogen and host immune system in TB [4–6], much of this information has been derived from studies in animal models or from adults with pulmonary TB (PTB). Measurement of gene expression changes following initiation of treatment in adults with PTB have demonstrated rapid changes in transcriptional signatures in whole blood within the first one to two weeks after commencing treatment [7, 8]. The initial down regulation of expression of inflammatory mediators has been coincident with rapid killing of actively dividing bacilli, while delayed changes in different networks of genes were coincident with resolution of lung pathology [8]. Other studies have demonstrated that the distinct TB transcriptional signature in acute disease reflects both altered gene expression as well as changes in cellular composition with a coincident reduction in T cell numbers [9, 10]. Remarkably little is known about how the developing immune system in young children responds to infection with *M*. *tuberculosis* [3] and whether it is distinct from, or similar to, that observed in adults. Childhood TB is often a “silent” infection, presenting insidiously and without the intense inflammatory response seen in other acute bacterial infections [1, 11], thus making diagnosis difficult. Indeed in Africa, children with TB are often only diagnosed post mortem [12].

The insidious presentation in children [3, 11] frequent association with tuberculin skin test anergy, and the high rate of extrapulmonary dissemination [1, 2] suggests an underlying failure of the immune system to both recognise and respond to infection. However, the immunological mechanisms responsible are incompletely understood [3].

Host gene expression profiling is particularly well-suited as a tool for studying the immunological mechanisms of childhood disease as it requires very small volumes of blood, little immediate sample processing and allows interrogation of multiple components of the immune system from a single sample [13–17].

To define the generalised immunological features in childhood TB, we studied temporal patterns of genome-wide RNA transcript abundance in the peripheral blood of children with TBM. We validated the findings in a second cohort of children with both PTB and TBM. We mapped the differentially expressed genes to known biological pathways, and evaluated T-cell functional responses in a third paediatric TB cohort of PTB, other extrapulmonary TB (EPTB—disease which has disseminated to bone, lymph node, renal or other organs aside from the brain) and TBM.

## Methods

### Cases and controls

The study was conducted at two major children’s hospitals, Tygerberg Hospital and Red Cross Children’s Hospital, in Cape Town, South Africa. The Western Cape has one of the highest incidences of childhood TB in the world and both hospitals have specialist research teams experienced in the management of TB in children. To avoid the confounding effect of HIV, only HIV negative children were included in the study. We studied three cohorts of cases and their respective controls. (Table A in S1 File and Figure A in S2 File).

In a region where TB is endemic and the annual incident rate is over 700/100 000, the choice of controls is always difficult. All children in South Africa receive BCG vaccination at birth; naive controls of young age may show changes in RNA expression as a secondary effect of immunisation, or of unrecognised TB exposure. In cohort 1, the choice of well, fully treated past history TB patients as controls eliminated bias of TB exposure and immunisation and allowed comparison of active disease with recovery. In cohorts 2 and 3 we aimed to establish how acute disease differs from children who are clinically well and either have evidence of TB infection but no disease (Mantoux >10mm and well with a normal chest radiograph) or no evidence of exposure and infection (well and Mantoux negative).

#### Controls

All controls were recruited over the same time period as cases, and were from the same geographic area in the Cape Town Metropole.

Controls for cohort 1 were identified from hospital records of paediatric patients treated for PTB and <14 years at time of recruitment. Controls for cohorts 2 and 3 were neighbourhood controls living within three streets of, and unrelated to, the case. All were healthy with a normal physical examination at the time and blood samples were collected prior to the completion of Mantoux testing. Exclusion criteria were a past history of TB treatment, age >14 years, prolonged treatment with steroids or other immunosuppressive drugs.

**Cohort 1 (discovery cohort)**—included 9 children with TBM studied sequentially at 0, 1, 4, and 6 months after their acute presentation. Based on time course analysis of other data sets, this cohort of well-studied patients with sequential time points, albeit small, was expected to produce statistically robust changes in expression over time. Diagnosis of TB was confirmed by two independent clinicians experienced in childhood TB (JFS and CH) based on a combination of history, examination, characteristic cerebro-spinal fluid (CSF) findings, culture of *M*. *tuberculosis* from sputum, CSF or gastric aspirate and neuro-radiological findings typical of TBM [18]. Culture negative cases were only included if CSF findings, neuro-imaging, and response to treatment were typical of TBM, and all other common infectious and inflammatory processes had been excluded. Controls were 9 healthy children at least 1 year after full completion of treatment for active PTB.

**Cohort 2 (confirmation cohort)—**were children studied only at the time of diagnosis with TBM (n = 13) or PTB (n = 28). Cases used were those where diagnosis was confirmed by two independent clinicians for both PTB (STA, CH) and TBM (JFS and CH). All cases fulfilled the WHO criteria for definite or highly probable childhood TB. Controls were 14 healthy, Mantoux negative children with no previous history of TB infection.

**Cohort 3 (T-cell functional studies)**—T-cell function (in whole blood) was studied sequentially (see supplementary methods) and comprised 36 children with acute TBM, 59 with acute, other EPTB and 57 with active primary PTB. Controls were 75 children with evidence of exposure and infection with *M*. *tuberculosis* (Mantoux ≥10mm, but no clinical disease). All cases enrolled fulfilled the WHO criteria for TB, with diagnoses confirmed by two independent clinicians (STA, BE) and were further categorised as suffering from PTB, other EPTB, or TBM based on CSF findings, imaging or culture of the organism from non-pulmonary sites.

All patients were enrolled following written informed parental consent and with the approval of the local hospital and university Research Ethics Committees of St Mary’s Hospital London and the University of Cape Town (EC 02/225; CT 013/2000).

#### RNA expression profiling and analysis

Peripheral blood collected into PAXgene tubes (PreAnalytiX) was frozen at -80°C for samples in both cohorts 1 and 2. RNA was extracted, linearly amplified and competitively hybridised with Stratagene Universal reference to a cDNA spotted microarray (Stanford Lymphochip) containing 38 000 genetic elements representing 18 000 unique human genes [19]. Full details for hybridisation, scanning and quality control have been described previously [15, 20] and are detailed in the online S1 Methods section.

#### Statistical methods: Time course analysis

We analysed serial gene transcript abundance in the peripheral blood from a cohort of children (cohort 1) with the most severe form of disseminated TB i.e. TBM, from presentation until completion of treatment at six months—the WHO standard treatment regimen for TBM at the time of this study [21]. Temporal analysis of gene expression from diagnosis until recovery post treatment was performed in order to identify disease-associated, differentially expressed genes. Time-course rather than single point analysis was chosen in order to control for individual genomic background variability. To account for the temporal and between-individual variation in RNA expression levels, the temporal behaviour of each unique probe on the microarray was modelled using a linear mixed-effects model. This model allows interpolation of individual time points and prediction of missing observations, summarising each probe with a fitted line that describes the mean changes in expression levels over time across all individuals. Statistically significant probes can then be detected using the slopes of these mean lines as test statistics. Full details are given in the supplementary web methods. The controls from cohort 1 were used as a visual reference for the cases in the time course analysis.

#### Statistical methods: Confirmation cohort

To validate the results of the time course analysis, we compared the admission time point of cohort 1 cases, with the PTB and TBM single time point (admission) cases in cohort 2. To define RNA expression in TBM and PTB as up- or down-regulated, we compared the mean expression level in TBM and PTB with the mean expression in the healthy, Mantoux negative controls using a logistic model and t–test. In order to confirm that the direction of gene regulation in TB relative to both the time course controls (healthy children with previous PTB) and single point cohort 2 controls were the same, we compared the mean expression of TBM cases in cohort 1 at day 180, with the cohort 1controls. As shown in Figure B in S2 File, the TB cases after treatment were concordant in direction of expression with healthy controls.

#### Biological pathway analysis

Significantly differentially expressed (SDE) genes were analysed using Ingenuity Pathways Analysis (IPA) (www.ingenuity.com) to give the biological functions and pathways represented in the dataset. A Fisher’s exact test with a Benjamini-Hochberg multiple testing correction was used to test if there was an association between the SDE genes and the pathway/biological function, and whether this was due to chance alone. Pathways were also assessed using the ratio of the number of SDE genes that mapped to a pathway divided by the total number of genes that existed in the canonical pathway.

#### Validation by quantitative RT-PCR

Differentially expressed genes identified by the microarray analysis were selected for confirmation by RT-PCR in paired admission and 6 month time point samples from cohort 1 (n = 8). Details of the genes, primers and experimental protocol are detailed in the online data supplement.

#### Functional T cell assays

T-cell proliferative and interferon gamma (IFNγ) responses to phytoheamaglutinin (PHA) stimulation were measured using the incorporation of tritiated thymidine and by measurement of IFNγ by ELISA respectively, as previously described [22] and detailed in the on-line supplementary methods.

#### Cell deconvolution

Celltype COmputational Differential Estimation (CellCODE), which computes the relative differences in cell proportions was used in order to assess to what extent the perturbation in gene expression could be due to changes in cell numbers [23]. Using the IRIS dataset as the reference dataset [24] we applied the CellCODE method to cohort two (TBM, TB and HC), and we retrieved cell proportions represented in surrogate proportion variables (SPVs).

#### Role of the funding source

There was no role played in this research by the funding source.

## Results

### Temporal change in gene expression in TBM (Cohort 1)

**Fig 1:**
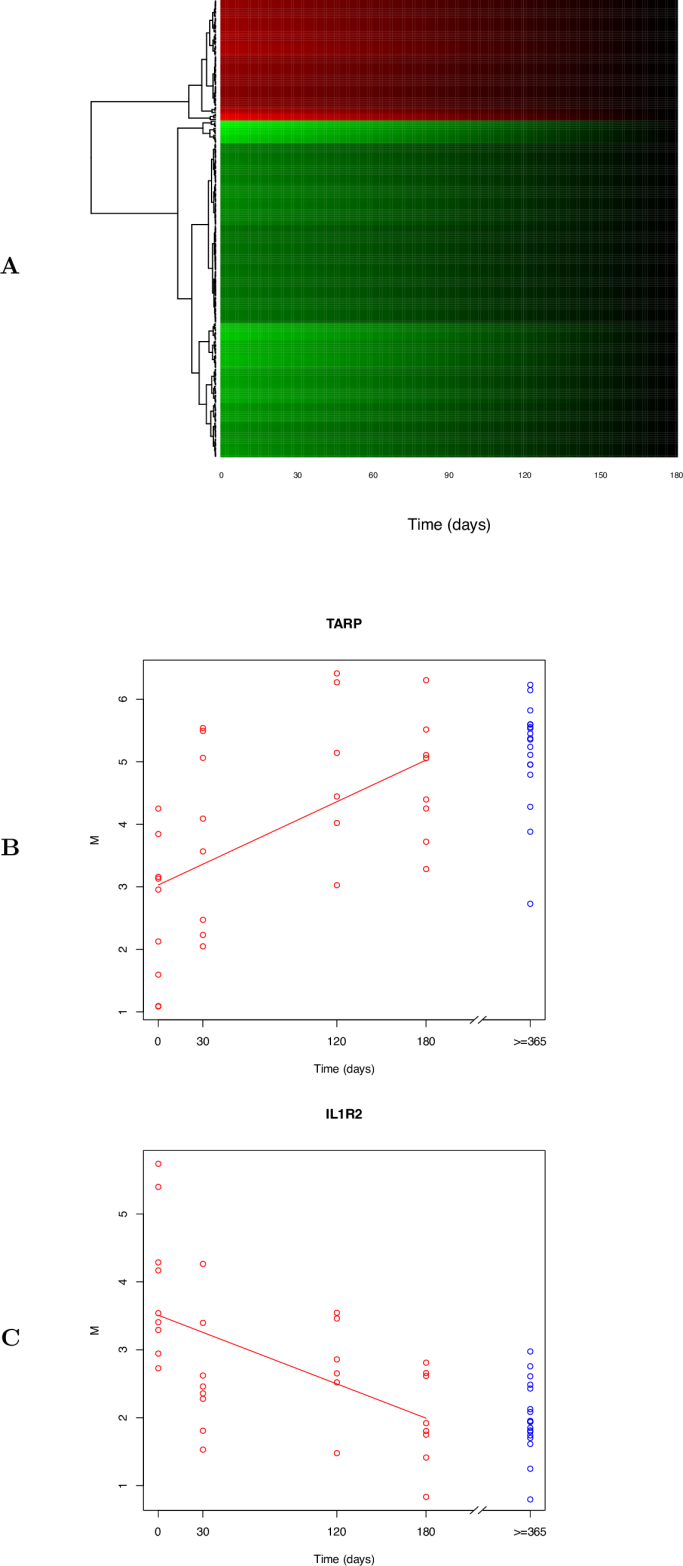
Modelled temporal changes in gene expression. **A**. Heat map showing modelled changes in expression of the significant gene transcripts in TBM patients from the time of diagnosis (0) to 180 days. Green represents lower transcript abundance, red represents higher transcript abundance and black represents no difference in expression as compared to healthy children with a past history of TB sampled at least one year after diagnosis and treatment. The relative degree of transcript abundance is indicated by the colour intensity derived from the fitted mean expression levels over time (see methods). Genes showing similar temporal patterns of expression have been clustered together. The apparent linear change in colour is derived from the statistical model that interpolates the observed time points and can therefore be represented as a continuum. **B and C**. Example plots of two significantly differentially expressed gene transcripts. Expression levels for each TBM patient (red circles n = 9) are shown from diagnosis (time 0) to day 180. Blue circles are expression levels for healthy children (n = 9) with a past history of TB sampled at least one year after diagnosis and treatment. M = “minus” and denotes the log_2_ ratio of the red and green channels. The line represents the fitted mean gene expression level over time, from linear mixed-effects model (see methods). 1b = *TARP*; 1c = *IL1R2*.

Analysis by linear mixed effects model [25] for gene transcript abundance in cases with TBM, over the 180 day time course from presentation to recovery, found 262 transcripts showing significant differential expression (SDE) between the admission and 6 month time point. At this 6 month time point, expression was also within the range of healthy children at least 1 year after active TB infection was diagnosed and fully treated. After filtering for duplicate transcripts, 204 unique transcripts mapped to 165 genes of known function. The majority, 140 transcripts (mapping to 110 known genes) (68%), were less abundant at the time of presentation with acute disease than they were at the time of recovery, or in the controls (healthy children with a past history of PTB at least 1 year after full completion of treatment). Only 64 transcripts (mapping to 55 known genes) were more abundant at the time of acute disease. A full list of SDE transcripts are shown in the online data supplement Table B in S1 File. Remarkably in the cases within 6 months (180 days) of commencing treatment, the differentially expressed transcripts returned to the levels of expression seen the control children, regardless of the direction of regulation. A graphical representation of this modelled dataset is shown in Fig 1A. Examples of the plots showing changes in transcript abundance are shown for two genes: *TARP*, increasing in abundance over time, and *IL1R2*, decreasing in abundance over time (Fig 1B and 1C).

### Confirmation of the repressive effects of TB infection on gene expression (Cohort 2)

**Fig 2:**
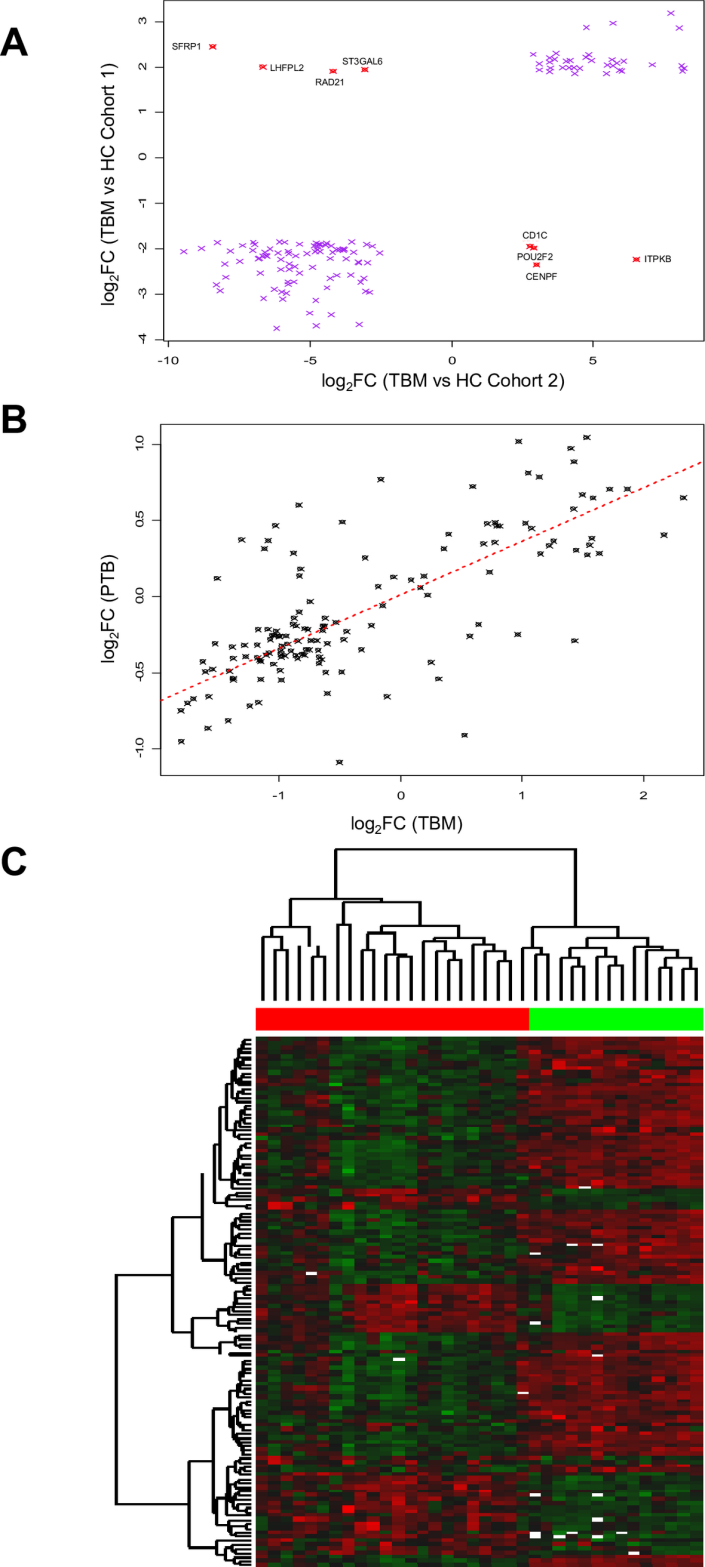
Confirmation of significantly differentially expressed genes from Cohort 1 in Cohort 2. **A**. Average log fold change in the SDE transcripts identified in the time-course study (cohort 1) and their corresponding log fold change in the single time-point study (cohort 2). 140/262 transcripts identified in cohort 1 were measured in cohort 2. 129 transcripts followed the same regulation pattern (purple crosses); and 11 showed opposite regulation (represented by red crosses, annotated by gene symbol). Correlation coefficient was r2 = 0.78, 95% CI = [0.71, 0.82] p<2x10-16. The y-axis shows log fold change of SDE gene transcripts in cohort 1 relative to cohort 1 Healthy Controls (HC), and the x-axis shows their log fold change in cohort 2 relative to cohort 2 HC. **B**. Average log fold change in TBM patients relative to cohort 2 HC (x-axis) plotted against average log fold change in PTB patients relative to cohort 2 HC (y-axis) of the significant transcripts (140) that were identified in cohort 1 and common to both cohorts. Least-squares fitted line is shown in dashes. Correlation coefficient was *r*^*2*^ = 0.71, 95% CI = [0.62, 0.79] *p*<2x10^-16^. **C.** Heat map showing almost complete discrimination between TBM cases from cohort 1 and cohort 2 and healthy controls (cohort 2) using 129 transcripts significantly differentially expressed in both cohorts. Gene list is provided in Table C in S1 File. Hierarchical clustering was performed by the complete linkage method to identify similar clusters. Solid red bar (top) shows cases, green bar shows controls. Intensity of colour indicates degree of reduced (green) or elevated (red) abundance of each transcript relative to healthy controls. White indicates no expression.

To validate our initial findings and establish if differentially expressed genes in acute TBM were also differentially expressed in other forms of TB, we compared the admission time point in the time course cohort (cohort 1), with the acute admission samples in a second cohort of children with TBM or PTB. We found a high degree of concordance of the direction of differential expression in the time course patients of cohort 1 and the single time point patients with TBM (Fig 2A), and PTB (Fig 2B) from cohort 2. As cohort 1 and 2 were analysed on different print runs of the Stanford Lymphochip array, not all transcripts were represented on both array batches. From the 262 SDE transcripts identified in the time course study, 140 transcripts were also represented on the single time point arrays used in the confirmation cohort. Out of these 140 transcripts, 129 were also significantly differentially expressed in the TBM single point cohort (i.e. cohort 2), and these allowed nearly complete discrimination between TBM cases from both cohorts and healthy, Mantoux negative controls (Fig 2C and Table C in S1 File). Although the direction of expression of these significant transcripts was similar in the PTB patients, the magnitude of differential transcript abundance was less in PTB than in TBM, and there was often overlap of PTB cases with the expression levels in healthy, Mantoux negative children (Figure C in S2 File). This suggests that the perturbation of gene expression in peripheral blood relative to a baseline, health-associated state is more pronounced in disseminated disease than in PTB.

### Biological function and pathway analysis of significantly differentially expressed genes

**Fig 3:**
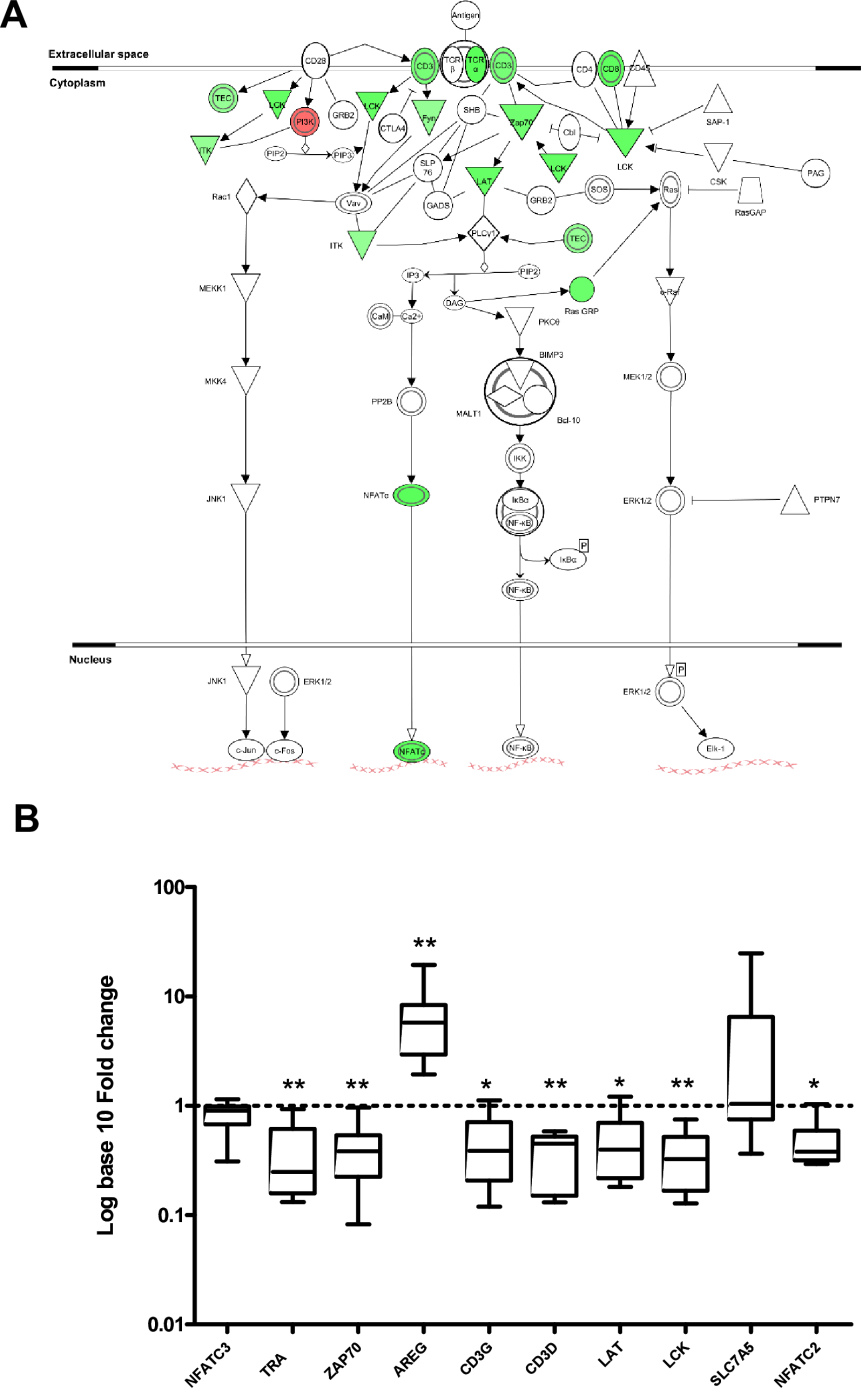
Gene expression of T-cell receptor signalling pathway and validation. **A**. Transcripts that were SDE in TBM patients at admission compared to the 6 month time point that mapped to the T-cell receptor signalling pathway. After activation of the T-cell receptor, a cascade of signalling events is initiated leading to gene induction. Gene products highlighted green are significantly less abundant in TBM patients at admission compared to the 6 month time point. Corrected *p* value on Ingenuity Pathways Analysis = 1.47E^-11^. Gene list provided in Tables D and E in S1 File. **B**. Validation of T-cell signalling pathway genes by RT-PCR in TBM patients (cohort 1). Selected genes in the T-cell signalling pathway were validated by RT-PCR including seven that were significantly less abundant at admission compared to post treatment (TRA, ZAP70, CD3G, CD3D, LAT, LCK, NFATC2) and one showing no change (NFATC3). Two genes were also included that were more abundant at admission compared to post treatment (AREG, SLC7A5) that acted as the positive controls. Fold change between TBM patients at admission and post treatment (n = 8) are shown relative to Beta actin control. Boxes show 25^th^ and 75^th^ percentile. Whiskers show lowest and highest data point and horizontal lines show medians. * *p*<0.05, ** *p*<0.01 shows significance using paired Wilcoxon rank test.

To identify the immunological pathways whose associated functions might be altered by the observed changes in RNA abundance, we mapped the genes that were SDE in TBM patients between the admission and 6 month time point, to biological functions and pathways using Ingenuity Pathways Analysis (IPA). The most significant functions represented in the category “Inflammatory response” (Table D in S1 File) were those of “immune response” (*p =* 8.86E^-14^), “activation of leucocytes” (*p =* 2.67E^-11^), “activation of lymphocytes” (*p =* 2.57E^-09^) and “activation of mononuclear leukocytes” (*p =* 3.18E^-10^), of which the transcript levels for 35/40 genes, 25/27 genes, 20/20 genes, and 21/22 genes respectively, were less abundant at admission than at the 6 month time point. In the category “haematological system development and function”, top functions included “T-cell development” (*p =* 1.02E^-07^) and “proliferation of T- lymphocytes” (*p =* 1.14E^-07^) of which transcript levels for 20/20 genes and 16/18 genes respectively, were less abundant at admission than at the 6 month time point. Further analysis of the SDE genes identified enrichment of genes in several T-cell related pathways, including the T-cell receptor signalling pathway (Fig 3A, *p =* 1.47E^-11^), and the T-cell cytotoxicity pathway (*p =* 1.83E^-07^) (Table E in S1 File). Multiple genes with lower transcript expression were identified in the T-cell receptor signalling pathway (Fig 3A and Table E in S1 File), including components of the T-cell receptor and co-stimulatory molecules (CD3D, CD3G, TCRα) and downstream signalling molecules (LAT, LCK, ITK, Ras GRP and NFAT). Differential expression of genes in this pathway was confirmed by RT-PCR in the same cohort (Fig 3B).

### Confirmation of immune cell representation

To exclude the possibility that the genes with lower transcript levels reflected depletion of sub-populations of immune cells from peripheral blood, or compartmentalisation of immune cells within the lung, we compared the gene expression profiles of childhood TB with our previously reported expression profiles of separated populations of CD4 and CD8 T-cells, B-cells, monocytes and NK cells from peripheral blood of healthy donors [26]. As shown in Table F in S1 File, the genes with lower transcript levels in TBM represented <10% of normally expressed T-cell genes, whereas 92% of CD8 and 90% of NK cell genes were normally expressed. Furthermore, reported studies of T cells numbers in acute childhood TB have not identified global reduction in specific T-cell subsets [27]. In addition, in order to further exclude the possibility that the changes in expression were due to altered proportions of cells in the blood of children with TB, we used the bioinformatics tool Celltype COmputational Differential Estimation (CellCODE), which computes the relative differences in cell proportions from the RNA expression data (supplementary methods).

When we applied the CellCODE method to cohort two we identified altered proportions of both T cell and neutrophil populations (Figure D in S2 File). However, when these differences were included in the differential expression model for TBM vs HC, the changes in gene expression were not explained by the differences in cell proportions (supplementary methods and Table J in S1 File).

### Analysis of significantly differentially expressed genes for evidence of regulation by key cytokines

The changes in transcript abundance for T-cell associated genes identified in the TBM patients are likely to represent selective changes in transcript abundance levels within these cells. IFNγ and TNFα have been identified as key cytokines for protective immunity to mycobacterial infection [28–31]. To establish whether the gene expression pattern in children with acute TB included IFNγ and TNFα-inducible genes, the gene expression profile in our patients was compared with the genes induced in peripheral blood cells by IFNγ type 1 interferons (IFN) alpha, beta, and omega, and TNFα, and by IFNγ in specific cell subtypes. As shown in Tables, G and H in S1 File <5% of the IFN or TNFα inducible genes were included in the significantly differentially expressed genes in childhood TB, suggesting that disseminated TB was occurring in the absence of an IFN or TNFα response in our patients, highlighting the surprising “silence” of the expected immune response to mycobacterial invasion.

### Functional studies validating gene expression changes (Cohort 3)

**Fig 4:**
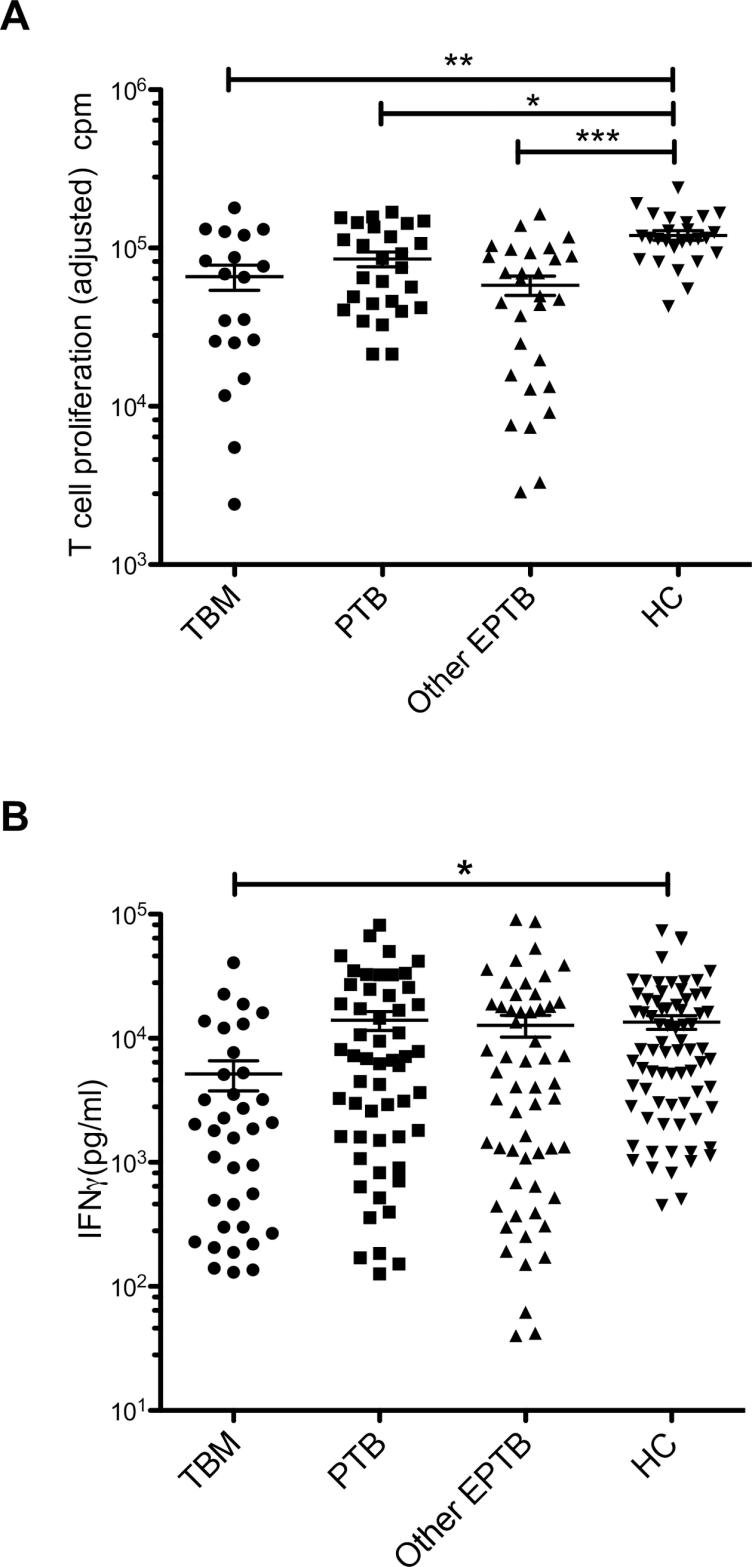
Functional T-cell responses in cohort 3. **A.** Adjusted T-cell proliferative responses (cpm) to PHA in acute TB (TBM n = 19, EPTB n = 29, PTB n = 27) and healthy Mantoux positive controls n = 26. Normalised proliferative responses were determined by deducting the value for the unstimulated well from that of the PHA well. Means are shown by horizontal bars together with standard error of the mean. Asterisk denotes significant differences in corrected p values. PTB vs HC * *p* = 0.018, TBM vs HC ** *p* = 0.001, EPTB vs HC *** *p<*0.0003. **B.** IFNγ production in response to PHA in acute TB (TBM n = 36, other EPTB n = 57, PTB n = 55) and healthy Mantoux positive controls (HC) n = 75. Medians are shown by horizontal bars together with their interquartile ranges. Asterisk denotes significant difference in corrected p value between TBM and controls * *p*<0.0003.

To establish whether the observed lower levels of expression of multiple genes was associated with impaired T-cell function, T-cell proliferative responses and IFNγ production in response to the mitogen PHA were studied in a third cohort of patients with PTB, other EPTB or TBM and compared with healthy, Mantoux positive childhood controls. As shown in Fig 4A, T-cell proliferative responses were significantly reduced on admission in all children with TB when compared to HC (TBM *p* = 0.001, other EPTB *p*<0.0003 and PTB *p* = 0.018) but with no differences between TB categories. Recovery of the impaired proliferative response occurred following treatment. Similarly, IFNγ production (Fig 4B) in response to PHA was depressed on presentation in patients with TBM compared to controls (*p<*0.0003), and recovered over time following treatment, but there was no significant difference seen with other EPTB or PTB compared to controls. Thus, the T-cell functional impairment parallels the changes in gene expression, overlapping that of HC in PTB and other EPTB and being most marked in disseminated disease with TBM.

## Discussion

Our genome-wide transcript analysis has revealed a predominance of reduced, rather than elevated gene expression in children with TB. We had expected to find evidence of a pro-inflammatory response (e.g. IL-1α, IL-1β, IL-6, IL-12) as mycobacteria are known to trigger inflammation through TLR2 and other pattern recognition receptors [32, 33]. Indeed, the belief that a pro-inflammatory state occurs in TBM has been the basis of attempts to ameliorate neuronal damage with steroids and other anti-inflammatory agents [34]. We also expected to find evidence of T-cell activation, and evidence of an IFNγ and TNFα response profile since patients present several weeks after primary infection, and long enough for T-cell and IFNγ responses to have developed [6]. Our findings of reduced T-cell responses, and absence of IFNγ and TNF response signatures is in keeping with the clinical impression that childhood TB is an immunologically “silent” disease in which mycobacterial invasion and dissemination occur without the expected host response. While the observed decrease in T cell proliferative responses could have been due to defective antigen presentation, we used a non-specific T cell mitogen, PHA, to measure proliferative and IFNγ responses which should, therefore, have been independent of antigen presentation. The inclusion of *M*. *tuberculosis* antigens could, therefore, have potentially shown differing responses. Similarly, a limitation of our analysis of functional responses is the lack of other cytokine measurements, with which to confirm the observed suppression of T-cell expression since it is well-documented that IFNγ responses are suppressed in acute TB [35].

Assignment of the differentially expressed genes to functional pathways revealed a remarkable pattern of reduced transcript levels for multiple genes required for T-cell activation, regulation of the cytotoxic granule mechanism, and surface proteins involved in T-cell homing and movement. This was mirrored by impaired responses of T-cells to mitogen. These results suggest that childhood TB is associated with an acquired immune defect, resulting from depression of multiple gene products required for an effective cellular response to the pathogen. Our findings of reduced expression of genes involved in T-cell cytotoxic responses provide an explanation for the reported reduction in serum granulysin concentrations in acute childhood TB [36]. These contrast with reported gene expression studies of adult TB in which up-regulation of pro-inflammatory genes has been observed [10, 37]. Berry et al [10] have previously reported an RNA expression signature of adult TB in which increased expression of IFNγ inducible genes and neutrophil genes was observed. Only 23 significantly differentially expressed genes in our childhood dataset were differentially expressed in the adult TB gene set of 312 genes (7.3%). This may reflect variation between the different arrays, but if real can be explained by the fact that childhood TB occurs after primary infection, and before T-cell immunity has developed and is distinct from adult TB, which generally presents as local pulmonary reactivation and is associated with marked inflammatory reaction. Further analysis comparing these findings to those in adults will be necessary to confirm that this is a distinct phenomenon in childhood tuberculosis and forms the basis of ongoing work.

Our findings have important implications for the understanding of the immunopathogenesis of childhood TB and of the severe forms of disseminated disease such as TBM. *M*. *tuberculosis* has evolved multiple strategies for evading the host immune response [32, 38–42] and our finding of repression of multiple genes involved in immune recognition of the mycobacteria and killing of infected cells may indicate an under-recognised, mycobacteria-mediated evasion strategy. We have observed the same reduced expression of key immune genes in both PTB and TBM, but with the magnitude of reduced expression being greater in TBM. In childhood, and particularly in very young children, the distinction between PTB and disseminated forms of the disease is not absolute, as many young children have features of military spread and organ involvement, and progression to extra pulmonary disease is much more common than in adults. Our finding of a gradation of gene expression in PTB and TBM suggest that those with disseminated forms of TB have a more severe impairment of the immune responses required to contain infection. Differences in mycobacterial load may also account for the differences in gene expression observed between patients with PTB and disseminated disease.

It has been well established that acute TB is associated with repressed IFNγ production [43] and mycobacteria are known to suppress HLA class II expression on infected monocytes through repression of *CIITA* and other genes involved in antigen processing and presentation [32, 44, 45]. Failure to develop an appropriate T-cell response following primary infection in children may explain the progression of disease and dissemination to brain and other organs. It is of interest that an “immune paresis”, similar to that in childhood TB, is also observed after critical illness and septic shock and, like TB, is associated with depressed T-cell mitogenic responses, reduced IFNγ production and cutaneous anergy [46]. While the mechanism underlying immune paresis in other severe infections is unknown, the reduced expression of multiple key genes in T-cell and other immune pathways that we have observed in TBM may represent a common mechanism underlying immune paresis in other acute illnesses. In order to assess if the observed changes in gene expression were due to changes in cell number we used a computational approach (CellCODE) to compute relative differences in cell population. While this showed a reduction in CD4 T lymphocytes in TBM, and more neutophils in PTB when compared to healthy controls, the differences in gene expression were not explained by the differing cell proportions, suggesting that the changes are due to repression of gene transcription, or rapid degradation of the RNA transcripts. Future studies will be needed to address the mechanism for the changes we have observed.

Further studies of gene expression in other clinical situations where immune paresis has been observed are needed to establish if reduced gene expression is a common mechanism for impaired immune function. We speculate that *M*. *tuberculosis* may be exploiting a common bacterial mechanism for down-regulating host responses as an immune evasion strategy enabling intracellular survival.

Our findings have relevance to the global effort to develop improved vaccines and treatment of TB. Further studies are needed to establish whether the reduced expression of host genes is mediated by specific host or bacterial transcription factors, or through a more widespread epigenetic process that silences multiple host gene.

## Supporting information

S1 Methods Contains additional methods. (DOCX)

S1 File Contains additional tables discussed above. (XLSX)

S2 File Contains additional figures discussed above. (DOCX)
