# Supplementary material for: Childhood tuberculosis is associated with decreased abundance of T cell gene transcripts and impaired T cell function
Source: PLoS One. 2017 Nov 15;12(11):e0185973. doi: 10.1371/journal.pone.0185973 (PMC5687722; doi:10.1371/journal.pone.0185973)
Supplement: S1 Methods — (DOCX) [file pone.0185973.s001.docx]

**Supplementary Methods**

**RNA profiling by microarray:** Total RNA extracted according to the standard PaxGene protocol was shipped frozen to Stanford University, California, USA. RNA quality and quantity was assessed using 1.25% Agarose denaturing gels, and a Genescan 2 spectrophotometer. Using an oligo-dT primer with a T7-RNA promoter, based on the modified Eberwine-Wang linear amplification procedure^2^, mRNA was transcribed into cDNA and linearly amplified. Three micrograms amplified antisense RNA was then reverse transcribed and labelled with Cy5-dUTP (Amersham) and combined with 3 μg of Cy3-labelled reference cDNA derived from a pool of RNA from a panel of 11 human cell lines (Stratagene Universal Human Reference RNA). The samples were washed and concentrated using Microcon YM30 columns (Amicon) and competitively hybridized on custom printed cDNA microarrays containing 37,632 elements from cDNA clones representing approximately 18 000 unique human genes (as previously described^3^). The hybridized slides were scanned using an AxonScanner II confocal microarray scanner (Axon Instruments). Comparative spot intensities were calculated from the images, and areas of poor quality excluded from further analysis using GenePix5.0 (Axon Instruments). The datasets discussed in this publication have been deposited in the Stanford Microarray Database, and NCBI's Gene Expression Omnibus and are accessible through GEO Series accession number GSE17762. Analysis was restricted to cDNA elements with a regression correlation of > 0.6, fluorescence intensities of greater or equal to 2.5 fold signal/background in Cy3 or Cy5 channels and a minimum signal intensity of greater than 100 in both channels for at least 80% of the arrays. Each array was normalized individually adjusting for plate, pin and intensity-dependent dye effects, and the scale adjusted so that the variance on each array was comparable.

**Time course analysis of gene expression data:** In order to account for both the temporal correlation and between-individual variation in RNA expression levels, the temporal profile of each unique probe on the microarray was analysed using a linear mixed-effects model. For each probe, the expression level for a particular individual was modelled as the sum of the following components: an intercept and a slope term describing the linear trend in the mean expression level for the probe over time; an individual specific term accounting for the individual’s deviation in RNA expression from the mean; and a term describing the measurement error. Estimates of the model parameters were obtained via maximum-likelihood estimation by employing an expectation maximization (EM) algorithm. Since the significance of the temporal variation is quantified by the slope of the mean line, hypothesis testing for this parameter was carried out to assess whether the slope was significantly different than zero. Given the small sample sizes, a permutation procedure was used to approximate the null distribution of this parameter and compute empirical p-values for each probe. The p-values were then corrected to account for multiple testing by controlling the false discovery rate at 10%. Expanded statistical method is included in the supplementary information. SDE for the discovery cohort was expressed as Log fold change of 0.88 using a corrected p value of with false discovery rate (FDR) of 10%

**Single time point analysis of gene expression data:** Data were filtered to include array elements that were present on more than 80% of the arrays. Within-slide normalization was performed through within-print-tip-group intensity dependent location normalization using the loess function, to correct for systematic variation introduced by overall spot intensity, dye and spatial effects. Scale normalization was applied to equalize the range of log2 ratios between slides. Differential expression analysis followed a “Two-Groups: Common Reference” experimental design and was performed using Linear models for microarray data analysis.^4^ Linear models were fitted and moderated *t*-statistics were calculated for each transcript. Adjustment for multiple hypothesis testing was performed via the Benjamini and Hochberg method that controls the FDR.^5^ Hierarchical clustering of the identified genes and samples as shown in the heat map of Figure 1A was performed using the complete distance as metric to identify the clusters.

**Analysis of the effects of changes in cell population on RNA expression:** In order to assess if the perturbation in gene expression could be due to changes in cell number, we used a computational approach: *in silico* cell deconvolution methodology. Recently developed cell deconvolution methods allow for estimation of proportions of multiple cell types in RNA expression data. This information can be included in the differential gene expression analysis to correct for changes in cell proportions. One such tool is Celltype COmputational Differential Estimation (CellCODE), which computes the relative differences in cell proportions.^6^ Using the Immune Response In Silico (IRIS) microarray dataset as the reference dataset,^7^ we included 5 key immune cell types in the estimation: neutrophil, monocyte, CD4+ T-cell, NK cell, and B-cell. CellCODE identifies marker genes specific to each cell type and calculates cell proportions (represented as surrogate proportion variables) (SPVs) for each of the 5 cell types and each sample. We applied the CellCODE method to the single time point samples (TBM, PTB and HC) (method= “raw” to get the SPVs and maximum 20 marker genes were allowed). We then included the SPVs in the differential expression analysis model in order to establish if the significantly differentially expressed genes between the patient groups were still significant after correcting for differences in cell proportions. Although we did indeed observe reduction in CD4 T cell and increase in neutrophil proportions in PTB and TBM, as compared to healthy controls, the differences in gene expression were not explained by the differences in cell proportions when the differences were included in the expression model. Table J in S1 file shows the corrected p value and the log2 fold change of the top 30 genes in the TBM vs HC comparison. Similar results were also observed when we applied the same approach to our previously reported dataset.^8^

**T-cell proliferative and interferon gamma responses:** In brief, 180 ul of heparinised whole blood, diluted 1:10 in tissue culture medium, was added to triplicate, 96-well, round-bottom tissue culture wells containing 20 uL of phytohaemagglutinin (PHA; Sigma, Poole, UK) at 5 ug/ml, or phosphate buffered saline as unstimulated control, and the plates incubated at 37^0^C in a CO_2_ incubator. On day 3, supernatants were removed from the wells containing PHA/blood (and replaced with an equal volume of tissue culture medium) and on day 6 from the unstimulated controls. Triplicate wells were pooled and stored at -80^0^C and IFNγ subsequently measured by enzyme-linked immunosorbent assay (ELISA) using antibody pairs from BD pharmingen (Becton-Dickinson, Oxford, UK).

T-cell proliferative responses were measured on day 4 of PHA culture with the addition of 1µCi of [methyl-3H] thymidine (Amersham Life Science, Little Chalfont, UK) to each well during the last 15 hrs of incubation and after harvesting of the supernatants. Tritium incorporation was measured by liquid scintillation and the average of triplicate counts calculated for each sample. Normalised proliferative responses were determined by deducting the value for the unstimulated well from that of the PHA well.

**Quantitative Real-Time RT-PCR validation:** RNA samples (acute and following treatment) from eight patients were reverse-transcribed to first-strand cDNA using Superscript III and random primers (Invitrogen). Real-time PCR was performed using Taqman technologies, Taqman Gene Expression Master Mix and the Step One Real-Time PCR System (Applied Biosystems). The primers were designed and manufactured using Assay-by-Design (Applied Biosystems) and are listed in Table I in S1 file. Reporter dye and quencher were FAM and NFQ, respectively. An initial input of 20ng per RNA sample was used. The threshold value of each gene was first normalized to the value of the constitutively expressed control gene *ACTB* (expression was unchanged in microarray analysis of T=0 to T=96). Gene induction or reduction values were calculated by comparing the normalized values of the acute vs. convalescent samples using the statistical formulation for ΔΔCt method (comparative Ct method) as described in “User Bulletin #2 ABI7700 Sequence Detection System” (Applied Biosystems).

All patients were enrolled following informed parental consent and with the approval of the local hospitals’ Research Ethics Committees.

**References:**

1. WHO. Global tuberculosis control. Geneva: World Health Organisation; 2011.
2. Eberwine, J. Amplification of mRNA populations using aRNA generated from immobilized oligo(dT)-T7 primed cDNA. Biotechniques 20, 584-91. (1996).
3. Alizadeh, A., Eisen, M., Davis, R. E. et al. The lymphochip: a specialized cDNA microarray for the genomic-scale analysis of gene expression in normal and malignant lymphocytes. Cold Spring Harb Symp Quant Biol 64, 71-8 (1999).
4. Smyth, G. K. Linear models and empirical bayes methods for assessing differential expression in microarray experiments. Stat Appl Genet Mol Biol 3, Article3 (2004).
5. Benjamini, Y. H., Y. Controlling the false discovery rate: a practical and powerful approach to multiple testing. Journal of the Royal Statistical Society Series B 57, 289-300 (1995).
6. [Chikina, M](https://www.ncbi.nlm.nih.gov/pubmed/?term=Chikina%20M%5BAuthor%5D&cauthor=true&cauthor_uid=25583121)., [Zaslavsky, E](https://www.ncbi.nlm.nih.gov/pubmed/?term=Zaslavsky%20E%5BAuthor%5D&cauthor=true&cauthor_uid=25583121)., [Sealfon, S.C](https://www.ncbi.nlm.nih.gov/pubmed/?term=Sealfon%20SC%5BAuthor%5D&cauthor=true&cauthor_uid=25583121). CellCODE: a robust latent variable approach to differential gene expression analysis for heterogeneous cell populations. [Bioinformatics.](https://www.ncbi.nlm.nih.gov/pubmed/25583121) 15;31(10):1584-91 (2015).
7. Abbas, A.R., Baldwin, D., Ma, Y., et al. Immune response in silico (IRIS): immune-specific genes identified from a compendium of microarray expression data. *Genes Immun* ;6(4):319-31 (2005).
8. [Anderson, S.T](https://www.ncbi.nlm.nih.gov/pubmed/?term=Anderson%20ST%5BAuthor%5D&cauthor=true&cauthor_uid=24785206),. [Kaforou, M](https://www.ncbi.nlm.nih.gov/pubmed/?term=Kaforou%20M%5BAuthor%5D&cauthor=true&cauthor_uid=24785206)., [Brent, A.J](https://www.ncbi.nlm.nih.gov/pubmed/?term=Brent%20AJ%5BAuthor%5D&cauthor=true&cauthor_uid=24785206). et al. Diagnosis of childhood tuberculosis and host RNA expression in Africa. N Engl J Med. 1;370(18):1712-23 (2014).
