# Supplementary material for: Childhood tuberculosis is associated with decreased abundance of T cell gene transcripts and impaired T cell function
Source: PLoS One. 2017 Nov 15;12(11):e0185973. doi: 10.1371/journal.pone.0185973 (PMC5687722; doi:10.1371/journal.pone.0185973)
Supplement: S2 File — (DOCX) [file pone.0185973.s003.docx]

Genome-wide RNA expression time course analysis

on children with TBM

Validate gene expression signature

on 2^nd^ cohort of acute TBM & PTB

Assess functional T cell responses in 3^rd^ cohort

of acute TB, EPTB and TBM pre & post treatment

Figure 1. Overview of the study and cohorts used


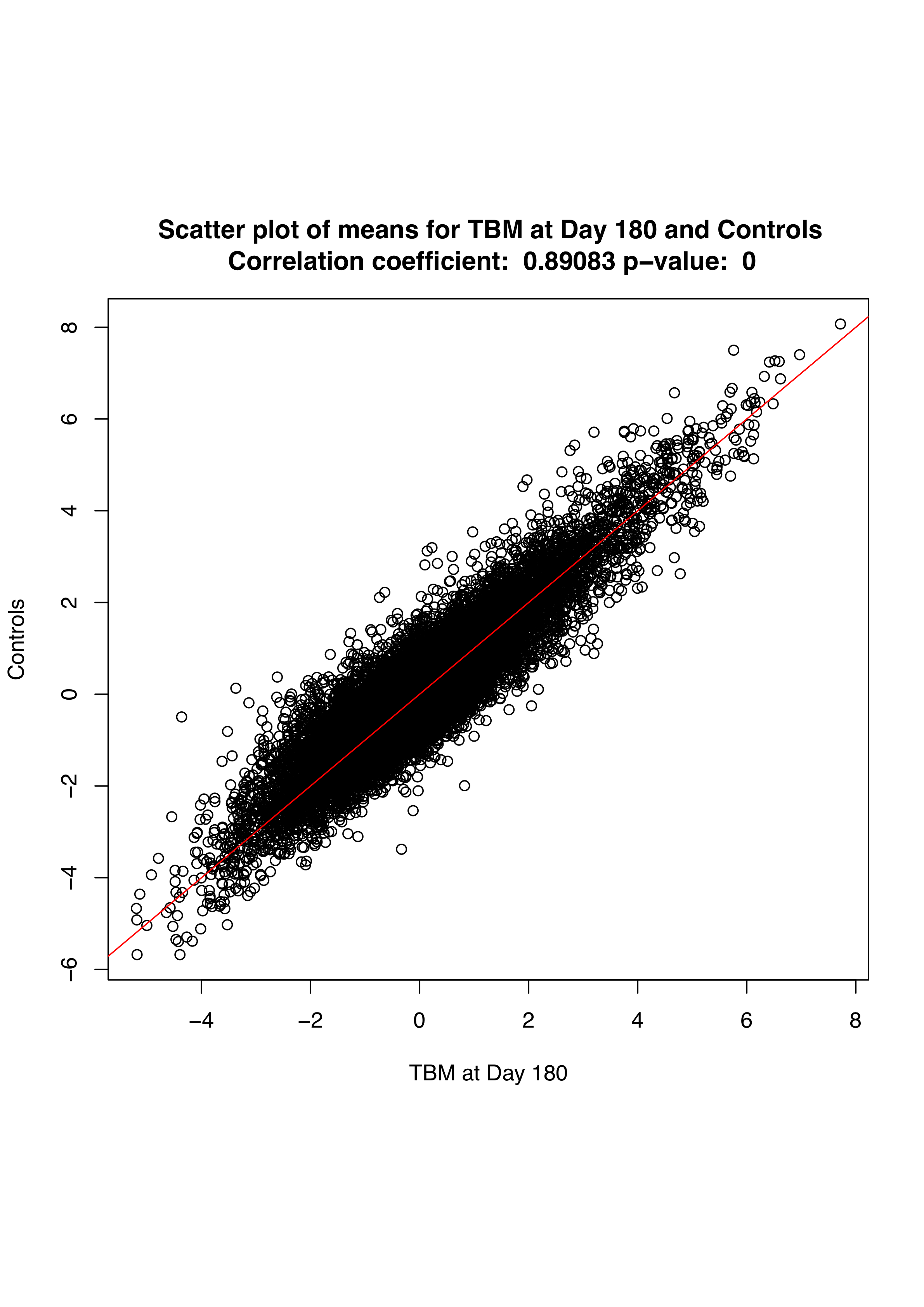


Figure 2. Comparison of mean expression of TBM cases in cohort 1 at day 180 with the healthy, cohort 1 controls (past history PTB cases at least one year after diagnosis and completion of full treatment).


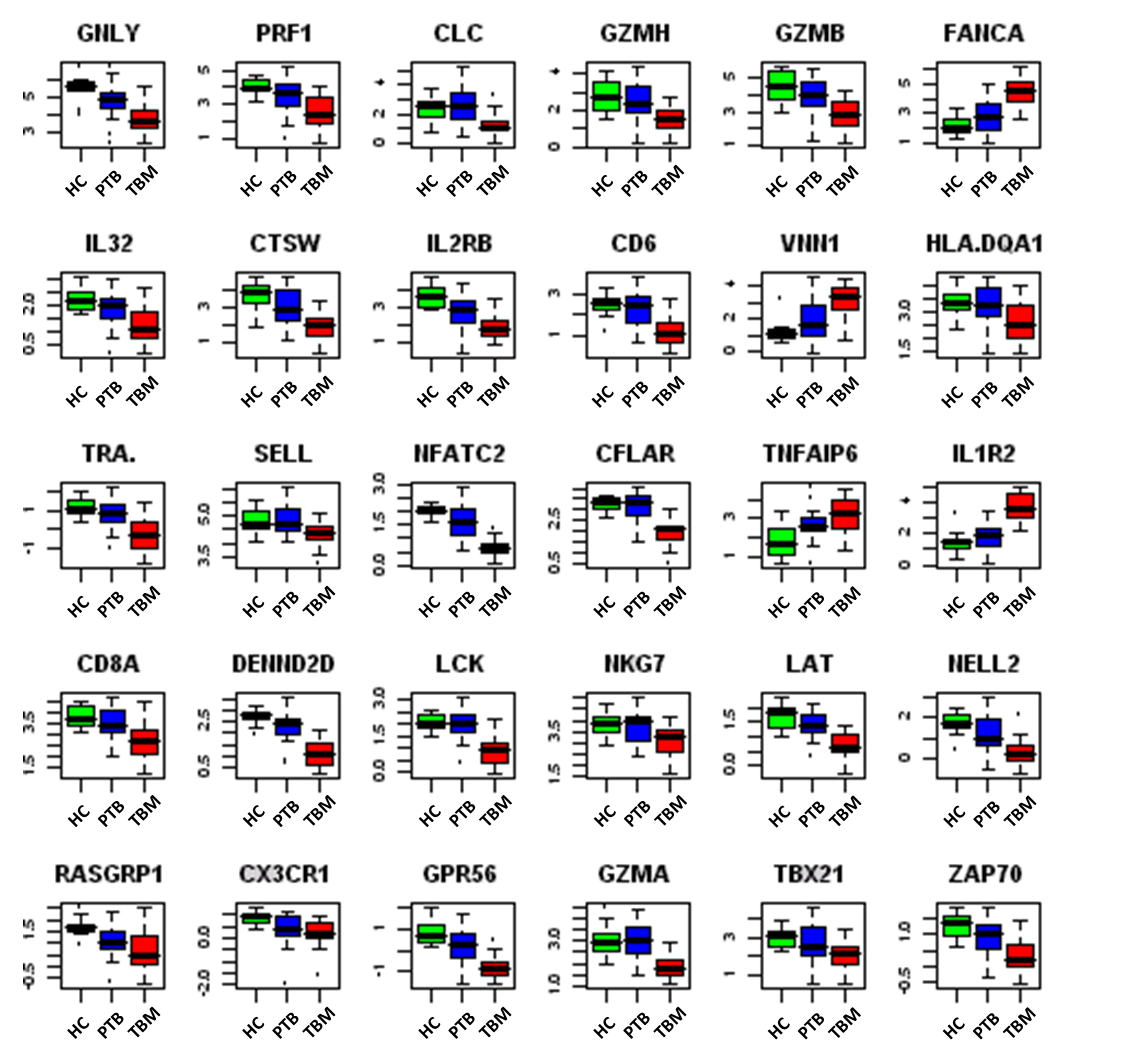


Figure 3: Box plots of gene expression for the top 30 significantly differentially expressed genes from Cohort 1 that were validated in Cohort 2. For significantly differentially regulated genes validated in cohort 2, the mean expression difference compared to healthy controls, was always less in PTB than in TBM. Furthermore, the significant genes identified in the TBM time course did not allow complete discrimination of PTB cases from healthy children. All genes had significant *t*-tests at the a=0.05 significance level after correction for False Discovery Rate. On each gene subplot, the expression of healthy controls (n=14) is shown in green, of PTB cases (n=26) in blue and of TBM cases (n=22) in red. Medians are depicted with a solid black line. The boxes indicate the 25% and 75% quartiles and the whiskers indicate the range of expression levels outside those quartiles. The y-axis shows log fold expression.


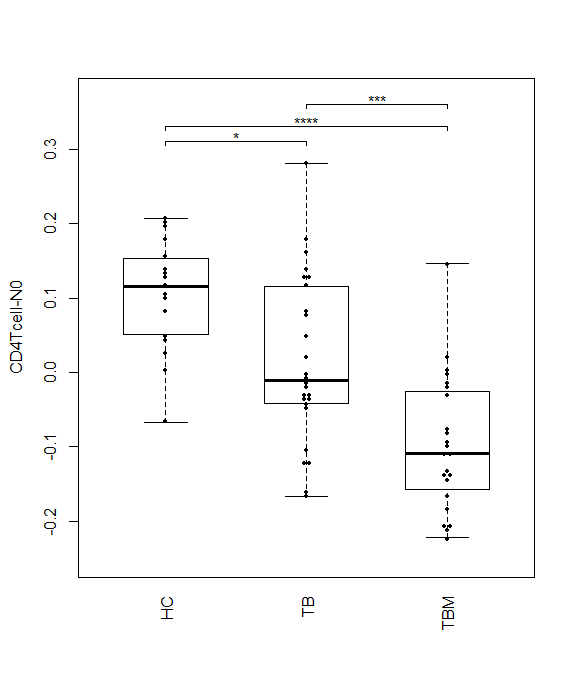

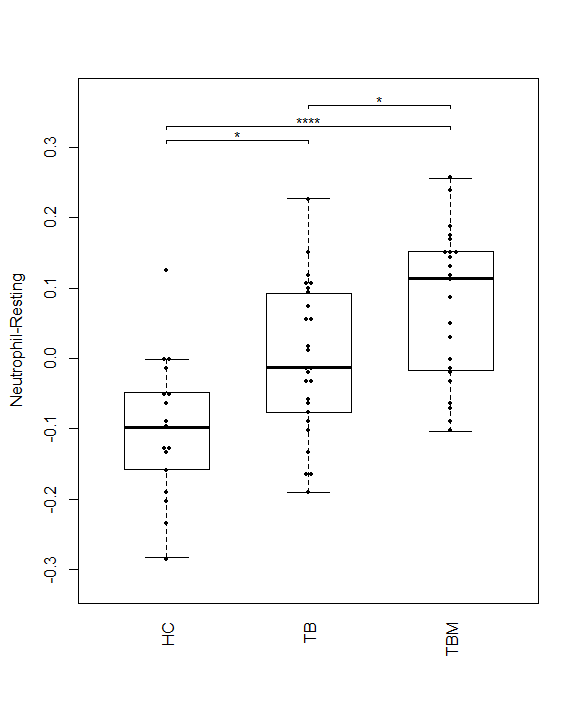


Figure 4: Box and whisker plots for the computed SPVs for CD4 T-cells and neutrophils for the 3 different groups of cohort 2 (HC, TB and TBM). Bonferroni corrected p values for multiple comparison obtained by pairwise Wilcox tests are indicated by asterisks on the figures. (*: p ≤ 0.05, ** p ≤ 0.01, *** p ≤ 0.001, **** p ≤ 0.0001)
